# Draft Genome Sequence of a Novel *Rhizobium* Species Isolated from the Marine Macroalga *Codium fragile* (Oyster Thief)

**DOI:** 10.1128/mra.00030-22

**Published:** 2022-05-02

**Authors:** Christopher H. Miller, Chanel Taylor, Jeremy G. Owen, Wayne M. Patrick, Chelsea J. Vickers

**Affiliations:** a Centre for Biodiscovery, School of Biological Sciences, Victoria University of Wellington, Wellington, New Zealand; SIPBS, University of Strathclyde

## Abstract

In the process of studying the relationship between marine macroalgae and their bacterial symbionts, we isolated a new species of *Rhizobium*, which we designated *Rhizobium* sp. nov. C1 (for “*Codium* 1”). Here, we report the complete genome sequence of *Rhizobium* sp. nov. C1.

## ANNOUNCEMENT

Oyster Thief (Codium fragile) is a green macroalga that is established worldwide and is becoming of increasing interest to researchers due to the identification of the high-value bioactive compounds it produces (1–5). We isolated and sequenced the genome of a bacterium associated with C. fragile that we subsequently identified as the Gram-negative *Rhizobium* sp. nov. C1.

The bacterium was isolated from C. fragile samples collected from a shoreline in Tauranga, New Zealand. Marine minimal seawater medium [0.5 M NaCl, 17 mM Na_2_HPO_4_, 11 mM KH_2_PO_4_, 4 mM (NH_4_)_2_SO_4_, 0.8 mM MgSO_4_·7H_2_O, and 0.3 mM CaC1_2_·2H_2_O] was supplemented with trace elements (6) and inoculated with C. fragile (10% [wt/vol]). The culture was grown in a shaking incubator (200 rpm) at 25°C. Cultures were subsequently subcultured every 2 to 5 days over a period of 2 weeks. Growth from liquid culture was spread onto BD Difco marine agar 2216. Repeated single-colony selection from marine agar was undertaken until a clonal strain was isolated, as determined by its uniform colony appearance and uniform growth rates upon restreaking. A stock of this strain was made by culturing the strain in BD Difco marine broth 2216 at 25°C, adding glycerol (25% [vol/vol]), and storing the strain at −80°C.

*Rhizobium* bacteria are of interest as important plant symbionts (7, 8), but their role as algal symbionts are less understood; therefore, we sequenced the genome of this new species. A single colony was used to inoculate 5 mL of Zobell marine broth, which was incubated for 15 h at 25°C with shaking. From this culture, genomic DNA was extracted using the Wizard genomic DNA purification kit (Promega) with the Gram-negative bacteria protocol. The library was prepared using in-house Tn*5*-mediated tagmentation (9), followed by PCR enrichment with KAPA HiFi HotStart ReadyMix (Roche). DNA fragments of 400 bp were size selected before 150-bp paired-end reads were generated on an Illumina HiSeq 4000 system by Genewiz (China). Other sequencing and assembly statistics are shown in Table 1.

**TABLE 1:** Assembly statistics and genome information for *Rhizobium* sp. nov. C1

Parameter	Finding for *Rhizobium* sp. nov. C1
Sequencing and assembly	
No. of Illumina reads	711,961
Total no. of Illumina bases	106,794,150
Avg coverage (×)	25
Genome description	
Genome size (bp)	4,240,398
G+C content (%)	61.6
Total no. of genes	4,022
Total no. of coding sequences	3,971
No. of proteins	3,928
No. of pseudogenes	43
No. of rRNAs	3
No. of tRNAs	44
No. of noncoding RNAs	4

Genome assembly was achieved using raw reads (no reads were trimmed or discarded on grounds of quality, which was verified with FastQC v.0.11.7 [10]). Reference assembly was carried out using SPAdes v.0.4.7 (11) with the Rhizobium ipomoeae reference genome ASM491216v1 (RefSeq assembly accession number GCF_004912165.1); this produced an assembly containing 22 contigs, with an *N*_50_ value of 469,644 bp. Genome annotation occurred through the NCBI Prokaryotic Genome Annotation Pipeline (PGAP) v.5.13 (12) as part of the GenBank submission process. All programs were run with default parameters unless otherwise stated. Genome features are summarized in Table 1.

The results of genome sequencing confirmed that we had isolated a species of *Rhizobium*, which we designated C1 (for “*Codium* 1”). The closest identified organism, based on average nucleotide identity (87.72%) determined using GTDB-Tk v.1.7.0 (13), was the plant-growth-promoting bacterium Rhizobium rhizophilum 7209-2 (RefSeq assembly accession number GCA_004912145.1) isolated from rape (Brassica napus L.) (7). The close relationship between *Rhizobium* sp. nov. C1 and this plant-growth-promoting bacterium indicates that *Rhizobium* sp. nov. C1 could be a marine algal symbiont; however, further investigation is required to validate this hypothesis.

### Data availability.

All data can be found under the NCBI BioProject accession number PRJNA773134, including raw reads (SRA accession number SRX13114500) and genome assembly information (GenBank accession number NZ_JAJGPW010000000).
